# Epigenetic Effects of Nanomaterials and Nanoparticles

**DOI:** 10.1186/s12951-020-00740-0

**Published:** 2021-01-06

**Authors:** Marta Pogribna, George Hammons

**Affiliations:** FDA/National Center for Toxicological Research, NCTR, HFT-110, 3900 NCTR Rd, Jefferson, AR 72079 USA

**Keywords:** Nanomaterials, Nanoparticles, Human cells, Epigenetic, DNA methylation, Histone modification

## Abstract

The rise of nanotechnology and widespread use of engineered nanomaterials in everyday human life has led to concerns regarding their potential effect on human health. Adverse effects of nanomaterials and nanoparticles on various molecular and cellular alterations have been well-studied. In contrast, the role of epigenetic alterations in their toxicity remains relatively unexplored. This review summarizes current evidence of alterations in cytosine DNA methylation and histone modifications in response to nanomaterials and nanoparticles exposures in vivo and in vitro. This review also highlights existing knowledge gaps regarding the role of epigenetic alterations in nanomaterials and nanoparticles toxicity. Additionally, the role of epigenetic changes as potential translational biomarkers for detecting adverse effects of nanomaterials and nanoparticles is discussed.

## Introduction

In recent years, the rise of nanotechnology has become an essential component of everyday human life and our environment [1]. Several online repositories, e.g. Nanodatabase [2], Nanowerk [3] and StatNano [4], list thousands of commercially manufactured nanotechnology products. Currently, nanoparticles (NPs), sized between 1 and 100 nm, and nanomaterials (NMs), a collection of nanoparticles having at least one dimension in the nanometer range, are widely used in household items, building materials, food and cosmetic products, sunscreens, water purification, toys, sports equipment, and medicine [1]. For example, coatings with silica dioxide nanoparticles (SiO_2_-NPs) and titanium dioxide nanoparticles (TiO_2_-NPs) are used to create self-cleaning, water-repelling, and heat-resistant surfaces; graphene nanoparticles and carbon nanotubes are widely used as composite materials to add strength with minimal weight to sporting equipment, such as tennis rackets, golf balls and clubs, and bicycles; silver nanoparticles (Ag-NPs) and copper nanoparticles (Cu-NPs), due to their strong antimicrobial properties, are widely used in clothing, linens, rugs, and towels, whereas platinum, palladium, rhodium, and cerium oxide nanoparticles are used in automobile catalytic converters to make vehicle exhaust less harmful.

Because of the widespread use of nanomaterials and nanoparticles, their biological effects on organisms and toxicity have been extensively studied over the past several years [5–9], however, their effect on the epigenome remains a developing area in the field of nanotoxicology research with limited and inconclusive data as well as many unanswered questions [10, 11]. This review summarizes the current knowledge about the effects of exposure to various nanomaterials and nanoparticles on two major epigenetic mechanisms, DNA methylation and histone modifications, and highlights the potential role of epigenetic changes in the mechanisms of nanomaterials and nanoparticles toxicity.

## Effect of nanomaterials and nanoparticles on cytosine DNA methylation

**Fig. 1:**
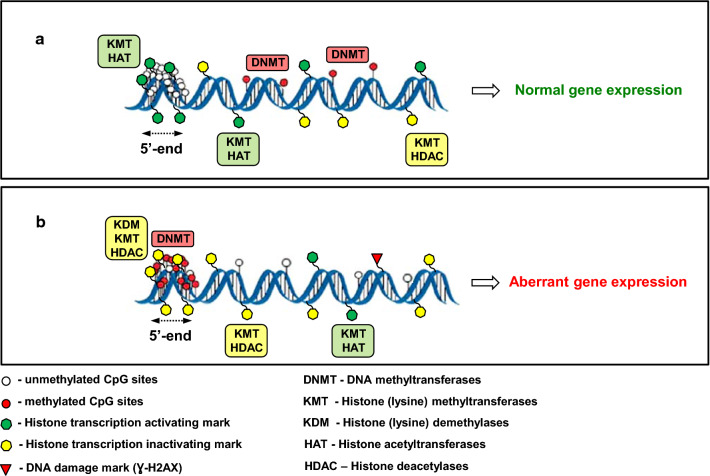
Epigenetic regulations in normal cells and after exposure to nanomaterials and nanoparticles. In normal cells, epigenetic mechanisms are well controlled and maintained by coordinated functioning of chromatin-modifying proteins, e.g., DNA methyltransferases, DNA demethylases, histone protein “writers” and “erasers”. In general, the epigenetic landscape of the normal genome consists of short unmethylated cytosine (*white circles*) enriched domains containing histone transcription activating modifications, including histone H3K4me3, H3K36me3, H3K9ac, and H3K27ac (*green hexagons*), predominantly located at the 5′-gene regions. These regions are embedded in a matrix of long methylated cytosine (*red circles*) domains containing both histone transcription activating (*green hexagons)* and transcription inactivating marks (*yellow hexagons)*. The accurate balance between these epigenetic modifications is critical for the proper maintenance of chromatin structure and gene expression. **a** Exposure to NMs and NPs reshapes the epigenetic genome landscape by increasing cytosine DNA methylation (*red circles*) and histone transcription inactivating modifications (*yellow hexagons)*, including increased histone H3K9 and H3K27 methylation, decreased histone K3K4 and H3K36 methylation, and loss of histone acetylation at the 5′-gene regions. Additionally, exposure to NMs and NPs causes damage to DNA (*red triangle*) across the genome and demethylation of cytosines (*white circles*) of previously methylated DNA domains. All these exposure-related events result in compromised chromatin structure and aberrant gene expression (**b**)

DNA methylation, a covalent modification of cytosine residues in DNA, is a major component of the cellular epigenetic regulatory mechanism (Fig. 1a), and one of the most studied epigenetic modifications. The DNA methylation reaction is the addition of a methyl group from *S*-adenosyl-l-methionine to carbon five of cytosine resulting in the formation of 5-methylcytosine (5-meC) in DNA [12]. Methylation of DNA is a dynamic and well-balanced process between DNA methylation and DNA demethylation reactions. DNA methylation is initiated and established by the family of de novo DNA methyltransferases DNMT3 (DNMT3A and DNMT3B) and is maintained during DNA replication by the maintenance DNA methyltransferase DNMT1 [13]. In somatic mammalian cells, methylation of DNA occurs solely at CpG dinucleotides. DNA demethylation is achieved through two different mechanisms (*i*) a “passive” replication-dependent mechanism during cell division and (*ii*) an “active” replication-independent mechanism. During active DNA demethylation, a family of ten-eleven-translocation (TET) proteins sequentially oxidizes 5-meC to 5-hydroxymethylcytosine (5-hmeC) and 5-carboxycytosine, which are later removed and replaced by cytosine via a base excision DNA repair mechanism [14].

Research in recent years has documented the effects of different types of nanomaterials and nanoparticles on DNA methylation (Fig. 1b) in various mammalian cells and mammalian organisms. Table 1 lists examples of changes in global and gene-specific DNA methylation induced by the exposure to well-known nanomaterials and nanoparticles in vitro and in vivo.

**Table 1:** Global and gene-specific DNA methylation alterations induced by various nanoparticles

Nanomaterials (NMs) and Nanoparticles (NPs)	In vitro or in vivo	Experimental design	Epigenetic effect	Reference
Carbon nanotubes (CNTs); single-walled and multi-walled carbon nanotubes (SWCNTs, MWCNTs)	In vitro	Human THP-1 monocytes exposed to 25 and 100 µg/mL of SWCNTs and MWCNTs for 24 h	No difference in global DNA methylation (5-mC) or DNA hydroxymethylation (5-hmC) Hypomethylation of 1127 genes, including *STAT5A, JAK3*-*STAT6, VEGFA, NOTCH1, NOTCH4, NOSS, WNT5B, PRKCZ, SH2D2A, SFRP1, FGFR1, TF, NAP2K2, AKT1, MEIS1*	[15]
CNTs: SWCNTs, MWCNTs	In vitro	Human bronchial epithelial cells 16HBE exposed to 25 and 100 µg/mL of SWCNTs and MWCNTs for 24 h	No difference in global DNA methylation (5-mC) or DNA hydroxymethylation (5-hmC) Hypomethylation of 2398 genes. Hypomethylation of individual CpG sites in 501 genes residing in the gene body and promoter regions, including *SKI, GSTP1, SHROOM2, NF1, AKP8L, FOXK2, EIF4E*	[16]
CNTs: SWCNTs, MWCNTs	In vitro	Human bronchial epithelial cells (16HBE) were treated with 0.25 μg/mL of MWCNTs or SWCNTs for four weeks (sub-chronic exposure) followed by two weeks of no particle exposure (recovery period)	No difference in global DNA methylation (5-mc) Hypermethylation of *HPCAL1*, *PRSS3, KLK3*, *KLF3* genes in SWCNT-exposed cells	[17]
CNTs: SWCNTs, MWCNTs	In vitro	Human bronchial epithelial cells 16HBE exposed to 25 and 100 µg/mL of SWCNTs and MWCNTs for 24 h	No difference in global DNA methylation (5-mC) or DNA hydroxymethylation (5-hmC) Differentially methylated for MWCNTs: *HDAC4, MAP3K10* Differentially methylated for SWCNTs: *MYO1C, NPAT/ATM, DNMT1* Differentially methylated for SWCNTs and MWCNTs: *PIC3R2* Hypermethylation of *ATM/NPAT* promoter	[18]
CNTs: SWCNTs, MWCNTs	In vitro	Human bronchial epithelial cells 16HBE exposed to 25 and 100 µg/mL of SWCNTs and MWCNTs for 24 h	MWCNTs induced significant dose-dependent loss of DNA methylation	[19]
Carbon dots (CDs)	In vitro	Human embryonic lung fibroblasts HEL12469 exposed to 10-500 µg/mL of positively or negatively charged CDs for 24 h	No difference in global DNA methylation (5-mC)	[20]
CNTs: MWCNTs	In vivo	Workers (n = 24) occupationally exposed to the MWCNTs and unexposed controls (n = 43) for 7 days	No difference in global DNA methylation (5-mC) or DNA hydroxymethylation (5-hmC) Changes at individual CpG sites in the promoter region of *DNMT1, ATM, SKI, HDAC4*	[46]
CNTs: MWCNTs	In vivo	C57BL*J*6 mice were exposed to 25 µl of MWCNTs suspension containing 50 µg of nanoparticles by a single oropharyngeal instillation, and solution was aspirated into the lungs. First effects were observed after 7 days, and mice were euthanized	Genomic hypomethylation in lung tissue and blood. Decrease in *EFN*-*ɣ* methylation *TNF*-*α* promotor hypomethylation Increase of methylation in *Thy*-*1* promotor	[38]
Carbon nanoparticles (CNPs)	In vivo	Adult zebrafish (*Danio rerio*) were randomly allocated into three separate tanks with 3 L of 0, 10 and 30 μg/mL CNPs suspensions for 60 days	Upregulation of *dnmt3b* and *Tet2* in heart tissue Dose‐dependent decreases in the mRNA expression of *dnmt1*, *dnmt3a,* and *Tet1* Promoter demethylation of *lebp, cd248b,* and *il11* Increase in transcriptions for *lebp* and *cd248b* in the high‐dose group	[40]
Modified Nano-Graphene quantum nanodots (M-GQDs)	In vivo	Zebrafish were exposed to 2, 10, and 50 mg/L modified reduced, hydroxylated, or aminated nano-graphene quantum dots for 7 days	Global DNA hypermethylation (tissue -specific and dose-dependent)	[41]
Laser printer-emitted nanoparticles	In vivo	Single intra-tracheal instillation of 2.5 mg/kg body weight of laser printer-emitted engineered nanoparticles into BALB/c mice: after 24 h, lung tissue was analyzed	Increase in global level of 5-meC by 25% and 5-hmeC by 50%	[39]
Silica NPs (SiO_2_-NPs)	In vitro	Human HaCaT cells exposed to 2.5-10 µg/mL SiO_2_-NPs for 24 h	Global DNA hypomethylation Dose-dependent decrease of the levels of DNMT1, DNMT3A, and methyl GpG binding protein 2 (MBD2)	[21]
SiO_2_-NPs	In vitro	Mouse Bhas 42 cells exposed to 15 or 25 µg/cm^2^ of the crystalline silica particles Min-U-Sil^®^ 5 for 48 h	~80% decrease in global DNA methylation Increase in the levels of DNMT3A and DNMA3B	[22]
SiO_2_-NPs	In vitro	Human HaCaT cells exposed to 10 µg/mL and 100 µg/mL of SiO_2_-NPs for 24 h	Reduced methylation of Alu repetitive elements	[23]
SiO_2_-NPs	In vitro	Human HaCaT cells exposed to10 µg/mL SiO_2_-NPs for 24 h	Promoter hypermethylation and decreased expression of PARP1 protein	[24]
SiO_2_-NPs	In vitro	Human bronchial epithelial cells BEAS-2B exposed to SiO_2_-NPs at 5 µg/mL for 30 passages (prolonged exposure)	Global DNA hypermethylation: 1973 hypermethylated CpG loci over 223 hypomethylated CpG loci. Hypermethylation of *CREB3L1* and *Bcl*-*2* gene promoters	[25]
SiO_2_-NPs	In vivo	31 workers from nanomaterial manufacturing and/or handling factories in Taiwan exposed to SiO_2_-NPs	Global DNA hypomethylation	[44]
SiO_2_-NPs	in vivo	White blood cells of 20 workers with long-term occupational exposure (mean time 14.5 years) to nanocomposite materials containing up to 20% SiO_2_-NPs	Long-term exposure caused changes in CpG methylation: 341 CpG sites hypomethylated 364 CpG sites -hypomethylated Short-term exposure did not affect DNA methylation patterns	[47]
Titanium dioxide nanoparticles (TiO_2_-NPs)	In vitro	Human MRC5 lung fibroblasts exposed to TiO_2_-NPs at 0.5 or 4 µg/mL for 24 and 48 h	Global DNA hypomethylation Decrease in total DNA methyltransferase activity	[26]
TiO_2_ -NPs	In vitro	Human lung epithelial cell line A549 exposed to silica or citrate coated TiO_2-_NPs and benchmark material P25 at 40 µg/cm^2^ for 48 and 72 h	Global DNA hypomethylation Demethylation of LINE1 repetitive elements	[27]
TiO_2_-NPs	In vitro	Human small airway epithelial cells exposed to TiO_2_-NPs at 0.5 and 30 µg/mL for 24 h	Demethylation of SINE B1 repetitive elements	[28]
TiO_2_-NPs	In vitro	Human cell lines: skin (A-431), lung (NL20), liver (HepG2), and colon (CaCo-2) exposed to TiO_2_-NPs at 100 µg/mL for 24 and 72 h	Global DNA hypomethylation in Caco-2, HepG2, and A-431 cells Increase in methylation of *CDKN1A, DNAJC15, GADD45A, GDF15, INSIG1, SCARA3, TP53,* and *BNIP3* genes Altered expression of *DNMT1, DNMT3A, DNMT3B*, *MBD2*, and *UHRF* genes	[29]
Gold nanoparticles (Au-NPs)	In vitro	Human kidney embryonic HEK293 cells exposed to 100 µg/mL of Au-NPs for 72 h	Global DNA hypomethylation	[23]
Au-NPs	In vitro	Normal human skin fibroblasts and human skin melanoma cells A375 exposed to Au-NPs at 62.5 µg/mL for 24 and 48 h	Global DNA hypomethylation	[30]
Au-NPs	In vitro	Human breast cancer cells SK-BR-3 exposed to Au-NPs at concentration of 3 µg/mL for 24, 48, and 72 h	No effect on global DNA methylation	[31]
Au-NPs	In vitro	Human hepatic HepG2 cells exposed to 10 µg/mL for 24 h	No effect on global DNA methylation	[32]
Au-NPs	In vitro	MRC5 human fetal fibroblasts exposed to 1 nM concentration of AuNPs for 48 and 72 h	No effect on global DNA methylation	[33]
Au-NPs	In vivo	Intratracheal administration of 5-, 60- and 250-nm Au-NPs at 2.5 and 0.25 mg/mL for 48 h to BALB/c mice	No effect on global DNA methylation and DNA hydroxymethylation Promotor hypermethylation in *Atm*, *Cdk*, and *Gsr* genes in mouse lung tissue Promotor hypomethylation in *Gpx* gene in mouse lung tissue	[42]
Silver nanoparticles (Ag-NPs)	In vitro	HT22 mouse hippocampal neuronal cell line exposed to 1-20 µg/mL of Ag-NPs for 48 h	Increase in DNA methyltransferases DNMT1, DNMT3A, and DNMT3B	[34]
Ag-NPs	In vitro	Human BEAS-2B cell line was exposed to 1 µg/mL Ag-NPs for 6 weeks	Marginal effects on DNA methylation: one differentially methylated gene promoter, corresponding to a gene (ENSG00000250358), 6 differentially methylated CpG sites and 5 differentially methylated tiling regions	[36]
Ag-NPs	In vitro	Human lung adenocarcinoma epithelial cells A549, exposed to 10-200 µg/mL Ag-NPs for 48 and 72 h	Global DNA hypermethylation	[35]
Ag-NPs Au-NPs Magnetite (Fe_3_O_4_) SPIONs	In vitro	Human hepatic cell line HepG2 treated with10 µg/mL Ag-NPs, 10 µg/mL Au-NPs, 5 µg/mL SPIONs for 24 h	No changes in promoter methylation of genes related to inflammation and apoptosis for any type of NPs studied	[32]
Zinc oxide nanoparticles (ZnO-NPs)	In vitro	Human MRC5 lung fibroblasts exposed to ZnO-NPs at 4 or 8 µg/mL for 24 and 48 h	Global DNA hypomethylation Decrease in total DNA methyltransferase activity	[26]
ZnO-NPs	In vitro	Human embryonic kidney cells HEK293 exposed to ZnO-NPs at 25 or 50 µg/mL for 48 h	Global DNA (5-mC) hypomethylation Increase in DNA hydroxymethylation (5-hmC) associated with increased expression of *TET1* and *TET2* genes	[37]
Copper (II) oxide NPs (CuO-NPs)	In vivo	Male BALB/c mice were exposed to a single intra-tracheal instillation of 2.5 mg/kg body weight of copper (II) oxide (CuO)-NPs and lung tissue collected after 24 h of exposure	Global DNA hypermethylation Reduced expression of DNA methyltrasferases, *Dnmt1*, *Dnmt3a*, and *DNMT3b*, and *Tet1*	[39]
CuO-NPs	In vivo	Female ICR mice were exposed to 8x10^5^ CuO-NPs in a whole-body inhalation chamber for either 3 days, 2 and 6 weeks, or 3 months	No changes in global DNA methylation	[43]

## DNA methylation alterations induced by exposure to nanomaterials and nanoparticles in vitro

### Carbon nanoparticles

Öner et al. [15] reported that exposure of human THP-1 monocytic cells to 25 and 100 µg/mL of single-walled carbon nanotubes (SWCNTs) and multi-walled carbon nanotubes (MWCNTs) for 24 h resulted in promoter hypomethylation of 1127 genes. In later studies, they reported a similar gene-specific cytosine DNA methylation response after exposing human 16HBE14 bronchial epithelial cells to SWCNTs and MWCNTs [16, 17]. Specifically, exposure to MWCNTs resulted in promoter hypomethylation of 2398 genes, whereas exposure to SWCNTs predominantly caused hypomethylation of individual CpG sites in 501 genes residing in the gene body and promoter regions, including the *SKI*, *GSTP1*, *SHROOM2*, and *NF1* genes. Significant changes in gene-specific DNA methylation were reported in another study after 24 h of exposure of 16HBE14 bronchial epithelial cells to SWCNTs and MWCNTs [18]. At least one CpG site was differentially methylated (while some of the CpG sites were hypomethylated, others were hypermethylated, often within the same gene promoter region) in *DNMT1* and *MYO1C* after exposure to SWCNTs, in *HDAC4* and *MAP3K10* after exposure to MWCNTs, and in *NPAT/ATM* and *PIK3R2* after exposure to both MWCNTs and SWCNTs. In contrast to alterations in gene-specific DNA methylation, no changes in the level of global cytosine DNA methylation were found in SWCNTs- and MWCNTs-exposed cells [16, 18]. However, in a study by Emerce et al. [19] the authors demonstrated a significant loss of global DNA methylation in 16HBE14 cells exposed to MWCNTs.

### Carbon dots

Carbon dots (CDs), one of the newer types of engineered NM, smaller than 10 nm but with a larger surface area. They are now widely used in medicine for drug delivery, bioimaging, and many other applications, but their potential toxicity and effect on epigenome are yet poorly understood. In a recent study by Sima et al. [20], the authors demonstrated that treatment of human embryonic lung fibroblast HEL 12469 cells, with various concentrations (10–500 μg/mL) for 24 h of positively or negatively charged CDs did not affect DNA methylation, even though the expression mRNA and miRNA were impacted.

### Silica nanoparticles

Treatment of human HaCaT cells with 2.5–10 µg/mL SiO_2_ nanoparticles (SiO_2_-NPs) for 24 h induced dose-dependent global DNA hypomethylation accompanied by reduction of DNMT1 and DNMT3A proteins [21]. A similar DNA hypomethylating effect of silica nanoparticles was found in two other studies [22, 23]. Seidel et al. [22] reported that treatment of mouse Bhas 42 cells with 15 or 25 µg/cm^2^ of the crystalline silica particle Min-U-Sil^®^ 5 resulted in a dramatic decrease, (~ 80%), in global DNA methylation after 48 h and this was accompanied by increased levels of DNMT3A and DNMT3B proteins. Sooklert et al. [23] found reduced methylation of Alu repetitive elements 72 h after exposing HaCaT cells to 10 µg/mL and 100 µg/mL of SiO_2_-NPs. Gong et al. [24] investigated the effect of SiO_2_-NPs on gene-specific methylation and demonstrated that the treatment of human HaCaT cells with 10 µg/mL SiO_2_-NPs for 24 h resulted in promoter hypermethylation and decreased expression of PARP1 protein.

Zou et al. [25] investigated changes in DNA methylation in response to prolonged exposure to silica nanoparticles. They reported that prolonged treatment, 30 passages, of human bronchial epithelial BEAS-2B cells with silica nanoparticles at a low non-cytotoxic concentration of 5 µg/mL resulted in marked DNA hypermethylation, which was evident by a predominant number (1973) of hypermethylated CpG loci over 223 hypomethylated CpG loci. Hypermethylation of the *CREB3L1* and *BCL*-*2* gene promoters was accompanied by significant down-regulation of gene expression.

### Titanium dioxide nanoparticles

Titanium dioxide nanoparticles (TiO_2_-NPs) are produced in large quantities and broadly used worldwide. The cellular and molecular effects, including genotoxic, of TiO_2_-NPs exposure have been extensively investigated, although there is a lack of conclusive information on their epigenetics effects. Several independent studies demonstrated that the loss of global DNA methylation is one of the major exposure-related epigenetic alterations caused by TiO_2_-NPs [26–29]. In particular, Patil et al. [26] showed that treatment of MRC5 lung fibroblast cells with 0.5 or 4 μg/mL TiO_2_-NPs for 24 and 48 h reduced the level of global DNA methylation. These DNA methylation changes were accompanied by a decrease in total DNA methyltransferase activity. In a separate study, Stoccoro et al. [27] investigated DNA methylation after exposure of human alveolar epithelial type-II-like A549 cells to silica- and citrate-coated TiO_2_-NPs. Exposure of A549 cells to 40 µg/cm^2^ of silica- or citrate coated TiO_2_-NPs for 72 h resulted in a loss of global DNA methylation, as evidenced by significant demethylation of LINE1 repetitive elements, with the strongest effect exhibited by citrate-coated TiO_2_-NPs. A similar demethylating effect on SINE B1 repetitive elements was found in human small airway epithelial cells (SAEC) after 24 h of exposure to 0.5 and 30 µg/mL TiO_2_-NPs, while no changes in the extent of LINE1 methylation were found [28]. Pogribna et al. [29] examined the effect of TiO_2_-NPs on global and gene-specific DNA methylation in several human cell lines: skin (A-431), lung (NL20), liver (HepG2), and colon (Caco-2). Cells were treated with TiO_2_-NPs at nontoxic doses for 24 and 72 h. Treatment with TiO_2_-NPs decreased global DNA methylation in Caco-2, HepG2, and A-431 cells, while methylation of the *CDKN1A, DNAJC15, GADD45A, GDF15, INSIG1, SCARA3, TP53,* and *BNIP3* genes increased in all four cell lines. Additionally, treatment with TiO_2_-NPs increased the expression of genes involved in establishing and maintaining DNA methylation patterns (*DNMT1, DNMT3A, DNMT3B*, *MBD2*, and *UHRF*) in a cell-type- and time-dependent manner, with the greatest effects found in NL20 and A-431 cells.

### Gold nanoparticles

Sooklert et al. [23] found reduced global DNA methylation 72 h after exposure of human kidney embryonic HEK293 cells to 100 µg/mL of gold nanoparticles (Au-NPs). Similar findings of DNA demethylating activity of AuNPs were reported by Patil et al. [30]; however, no changes to the extent of global DNA methylation were found in Au-NPs-treated SK-BR-3 human breast cancer cells [31], HepG2 human liver cancer cells [32], or MRC5 human fetal fibroblasts [33]. Likewise, no changes in the DNA methylation status of the down-regulated PROS1 gene were found in Au-NPs-treated MRC5 cells [33].

### Silver nanoparticles

Several studies have investigated the effect of Ag-NPs on cytosine DNA methylation. Mytych et al. [34] demonstrated that treatment of mouse hippocampal neuronal HT22 cells with 5 µg/mL Ag-NPs for 48 h induced marked genomic cytosine DNA hypermethylation and increased protein levels of DNMT1, DNMT3A, and DNMT3B. Importantly, these changes persisted for 144 h, with cytosine DNA methylation continuing to increase, after removal of Ag-NPs from the culture media. Blanco et al. [35] reported increased global cytosine DNA methylation in response to the exposure of human lung adenocarcinoma A549 cells to 200 µg/mL Ag-NPs for 72 h, while no changes were found in cells exposed to the lower concentrations of Ag-NPs ranging from 10 to 100 µg/mL. Brzóska et al. [32] reported similar findings with no effect of Ag-NPs on human liver cancer HepG2 cells, and Gliga et al. [36] demonstrated a minimal effect on DNA methylation in human BEAS-2B cells exposed to 1 µg/mL Ag-NPs for 6 weeks.

### Zinc oxide nanoparticles

Similar to TiO-NPs, the major exposure-related effect of zinc oxide (ZnO)-NPs is the loss of global DNA methylation. Treatment of MRC5 lung fibroblast cells with ZnO-NPs at concentrations of 4 or 8 μg/mL for 24 and 48 h markedly reduced the level of global DNA methylation [26]. These DNA methylation changes were accompanied by a decrease in total DNA methyltransferase activity. Likewise, a profound DNA demethylating effect of ZnO-NPs, evidenced by a marked decrease in the levels of 5-meC in DNA and locus specific-DNA hypomethylation, accompanied by the reduced expression of *DNMT1* and *DNMT3B* genes in human embryonic kidney HEK293 cells treated with 25 or 50 μg/mL of ZnO-NPs for 48 h was reported by Choudhury et al. [37]. Interestingly, treatment with 50 μg/mL of ZnO-NPs resulted in a substantial increase in the levels of 5-hmeC in DNA, which was associated with increased expression of the *TET1* and *TET2* genes.

## DNA methylation alterations induced by exposure to nanomaterials and nanoparticles in experimental animal models in vivo

### Carbon nanoparticles

Brown et al. [38] reported global DNA hypomethylation in lung tissue and white blood cells in C57BL/6 mice 7 days after a single otopharyngeal instillation of 50 µg of MWCNTs. Assuming that a mouse weighs 25–30 g, this dose would equal 1.7–2 mg/kg body weight. In addition to a decrease of global DNA methylation, they found decreased methylation in *Ifn*-ɣ and *Tnf*-*ɑ* genes. In contrast, Lu et al. [39] reported a significant increase in the global level of 5-meC by 25% and 5-hmeC by 50% in the lung tissue of male BALB/c mice 24 h after a single intra-tracheal instillation of 2.5 mg/kg body weight of laser printer-emitted engineered nanoparticles. They hypothesized that the elevation of 5-hmeC was associated with diminished expression of the *Tet1* gene which encodes a major TET1 methylcytosine-deoxygenase that sequentially converts 5-meC into 5-hmeC and then further into 5-formylcytosine and 5-carboxycytosine during active DNA demethylation. Interestingly, an elevation of 5-hmeC in the lung tissue of treated mice was accompanied by reactivation of major LINE1 and SINE B1 repetitive elements. In a recent report, Zhou et al. [40] demonstrated that prolonged (60 days) exposure of zebrafish (*Danio rerio*) to 10 and 30 µg/mL black carbon NPs (50 nm) markedly enhanced global cytosine DNA methylation, but reduced gene promoter methylation of *lepb, cd248b*, and *il11a* in heart tissue. Similar DNA methylation changes were recently reported by Hu et al. [41], who demonstrated that exposure of zebrafish to 2, 10, and 50 mg/L modified reduced, hydroxylated, or aminated nano-graphene quantum dots for 7 days resulted in dose-dependent and tissue-specific increase in global DNA methylation.

### Gold nanoparticles

Tabish et al. [42] studied the effects of different doses and sizes of Au-NPs on gene-specific methylation in mouse lungs. They found that a single intra-tracheal instillation of 60 nm Au-NPs to BALB/c mice induced hypermethylation of *Atm*, *Cdk*, and *Gsr*, and hypomethylation of *Gpx* in lung tissue 48 h after instillation. Additionally, there were differences in *Gsr* and *Trp53* methylation between low (0.25 mg/kg body weight) and high (2.5 mg/kg body weight) doses of Au-NPs, and differences in *Trp53* methylation relative to the nanoparticle size.

### Copper oxide nanoparticles

Exposure-related DNA methylation changes, characterized by DNA hypermethylation and concomitant reduced expression of DNA methyltrasferases, *Dnmt1*, *Dnmt3a*, and *DNMT3b*, and *Tet1* were found in the lung tissue of male BALB/c mice 24 h after a single intra-tracheal instillation of 2.5 mg/kg body weight of copper (II) oxide (CuO)-NPs [39]. In contrast, no changes in global DNA methylation, despite the marked transcriptomic changes, were found in female ICR mice exposed to 8x10^5^ CuO-NPs in a whole-body inhalation chamber for either 3 days, 2 and 6 weeks, or 3 months [43].

## DNA methylation alterations induced by exposure to nanomaterials and nanoparticles in humans

In contrast to more extensive experimental in vitro and in vivo studies on DNA methylation alterations caused by exposure to nanomaterials and nanoparticles, there is limited evidence on the effects of engineered nanomaterials and nanoparticles on DNA methylation in humans. Liou et al. [44] investigated the status of DNA methylation in white blood cells in 31 workers from nanomaterial manufacturing and/or handling factories in Taiwan exposed to SiO_2_-NPs. They found a significant decrease in global DNA methylation in SiO_2_-NPs-exposed workers compared to control individuals (n = 43). Importantly, this loss of global DNA methylation inversely correlated with the levels of 8-hydroxy-2′-deoxyguanosine (8-OHdG), a marker of oxidative DNA damage [45], in urine and white blood cells of exposed workers. In contrast, Ghosh et al. [46] did not find a difference in the levels of DNA methylation in blood cells in workers (n = 24) occupationally exposed to MWCNTs in the workplace compared to control individuals (n = 43); however, the authors found a significant change in methylation at individual CpG sites located in the promoter regions of the *DNMT1*, *ATM, SKI*, and *HDAC4* genes.

Recently, Rossnerova et al. [47] investigated global and gene-specific epigenetic DNA methylation in white blood cells in 20 workers with long term occupational exposure (mean time 14.5 years) to nanocomposite materials containing epoxide resin with up to 20% SiO_2_. They found that long-term exposure caused substantial changes in CpG methylation, in which 341 CpG sites were hypomethylated and 364-hypermethylated. In contrast, short term exposures did not affect DNA methylation patterns.

## Effects of nanomaterials and nanoparticles on histone modifications

In addition to alterations in DNA methylation induced by nanomaterials and nanoparticles, disruption of normal patterns of histone modifications is another epigenetic response. Histone modifications, posttranslational covalent modifications of the amino-terminal tails of histone proteins, including phosphorylation, methylation, and acetylation, are additional major components of the epigenetic regulatory mechanism (Fig. 1a). Like methylation of DNA, histone modifications are a dynamic process tightly controlled by the balance between “writers” and “erasers” [48]. “Writers”, including histone phosphorylases, acetyltransferases, and methyltransferases, introduce a particular chemical histone modification, whereas “erasers”, including histone phosphatases, deacetylases, and demethylases are responsible for removal of chemical modifications. Accumulated evidence demonstrates substantial disruption of the histone modification patterns as a result of exposure to nanomaterials and nanoparticles (Fig. 1b).

## Alterations of histone modifications induced by exposure to nanomaterials and nanoparticles in vitro

### Effect of nanomaterials and nanoparticles on phosphorylation of histone H2AX

One of the most consistent alterations induced by exposure to a broad range of nanomaterials and nanoparticles is increased phosphorylation of histone H2AX at serine-139 (ɣ-H2AX). It is well-documented that ɣ-H2AX is generated as a response to various types of DNA lesions and is one of the earliest DNA damage responses [49]. Table 2 lists examples of the ɣ-H2AX changes induced by exposure to well-known nanomaterials and nanoparticles in vitro and in vivo.

**Table 2:** Effect of various nanoparticles on histone H2AX phosphorylation

Nanomaterials (NMs) and Nanoparticles (NPs)	In vitro or in vivo	Experimental design	Epigenetic effect	Reference
SiO_2_-NPs	In vitro	Human intestinal CaCo-2 cells exposed to quartz, SiO_2_-55 nm or SiO_2_-15 nm at 4, 16, 32 and 64 μg/mL or 15 nm at 64 μg/mL for 24 h	Increase in ɣ-H2AX	[56]
TiO_2_-NPs	In vitro	Human lung adenocarcinoma epithelial cells A549 exposed to 50-1000 µg/mL or 1-100 µg/mL TiO_2_-NPs for 1, 24, and 48 h	Increase in ɣ-H2AX independent of oxidative stress	[51]
TiO_2_ -NPs and Nano-cobalt (Nano-Co)	In vitro	Human lung adenocarcinoma epithelial cells A549, exposed to 5-15 µg/mL TiO_2_-NPs and nano-Co for 12 h	Increase in ɣ-H2AX	[52]
TiO_2_-NPs	In vitro	Human lung adenocarcinoma epithelial cells A549, macrophage-likeTHP-1 cells, and human pulmonary microvascular endothelial cells HPMEC-ST1.6R were exposed to 5, 200, and 800 µg/mL TiO_2_-NPs for 4 and 24 h	Increase in ɣ-H2AX in THP-1 and HPMEC-ST1.6R cells	[53]
TiO_2_-NPs	In vitro	Human dermal fibroblasts isolated from neonatal foreskins, exposed to 100, 30, 10, 3, and 1 µg/mL TiO_2_-NPs for 24 h	Increase in ɣ-H2AX	[54]
TiO_2_ -NPs	In vitro	Human skin fibroblasts cell line (BJ), exposed to 10, 25, 50, 100, 250, 500, and 1000 µg/mL TiO_2_-NPs for 24 h	Increase in ɣ-H2AX	[55]
Ag-NPs	In vitro	Human lung adenocarcinoma epithelial cells A549, exposed to 10-200 µg/mL Ag-NPs for 48 and 72 h	Increase in ɣ-H2AX	[35]
Ag-NPs	In vitro	Human skin keratinocytes (HaCaT), human lung (A549) and human breast adenocarcinoma cells (MCF-7) treated with 1.0 µg/mL Ag-NPs for 4 h	Increase in ɣ-H2AX in A549 and MCF-7 cells.	[50]
Au-NPs	In vitro	Human MDA-MB-231 and MDA-MsB-468 breast cancer cells exposed to 100, 250, and 500 µg/mL positively (+) and to 250 and 500 µg/mL negatively (-) charged Au-NPs for 24 h	Increase in ɣ-H2AX	[57]
CuO-NPs	In vitro	Human hepatocellular carcinoma cells HepG2 (well-differentiated) and SK-Hep-1 (poorly differentiated) were exposed to Cu-NPs at 0, 10, 25, 50, 75, and 100 µg/mL for 24 h	Increase in ɣ-H2AX, especially in SK-Hep-1 cells, in dose–dependent manner	[58]
Cerium dioxide NPs (CeO_2_-NPs)	In vitro	Human peripheral blood lymphocytes were exposed to CeO_2_-NPs at 6, 12, and 18 µg/mL for 3-24 h	Increase in ɣ-H2AX	[59]
Arsenic trioxide NPs (As_2_O_3_-NPs)	In vitro	Human embryonic kidney HEK293 or HeLa cells were exposed to As_2_O_3_-NPs at 0.2–0.8 µM for 24, 48, and 72 h	Increase in ɣ-H2AX	[60]
ZnO-NPs	In vivo and in vitro	Chickens fed diets containing ZnO-NPs at 10–200 mg/kg for 24 weeks, then artificially inseminated, embryonic development monitored, and ovarian cells cultured	Increase in ɣ-H2AX	[61]
Iron oxide nanoparticles (S-ION) silica coated	in vitro	Human A-172 glioblastoma cells exposed to various concentration of S-ION (5-100 µg/ml) for 3 and 24 h	Increase in H2AX at 50 and 100 µg/ml S-IOP for 24 h	[62]

Several reports have shown that exposure of mammalian cells to Ag-NPs results in ɣ-H2AX induction. Zhao et al. [50] reported that treatment of human cancer cells with 1.0 µg/mL Ag-NPs for 4 h induced formation of ɣ-H2AX in lung adenocarcinoma A549 cells and human breast adenocarcinoma MCF-7 cells, but not in human skin HaCaT keratinocytes. Dose-dependent induction of ɣ-H2AX was found in A549 cells treated with 10-200 µg/mL Ag-NPs for 48 or 72 h [35]. Induction of ɣ-H2AX was also reported in A549 cells [51–53], human dermal fibroblasts [54], and human skin fibroblasts [55] treated with TiO_2_-NPs, and in human intestinal Caco-2 cells treated with SiO_2_-NPs [56]. Increased ɣ-H2AX has also been shown in human cells after exposure to Au-NPs [57], and other NPs, including copper oxide (CuO)-NPs, cerium dioxide (CeO_2_-NPs), arsenic trioxide (As_2_O_3_-NPs), zinc oxide (ZnO-NPs), and iron oxide NPs [58–62].

In the majority of these studies, increased formation of ɣ-H2AX was accompanied by the induction of oxidative stress; however, phosphorylation of histone H2AX independent of oxidative stress was also shown.

## Effect of nanomaterials and nanoparticles on histone phosphorylation, acetylation, and methylation

In addition to induction of ɣ-H2AX, exposure to nanomaterials and nanoparticles resulted in other types of histone modifications (Table 3).

**Table 3:** Changes in histone modifications and histone-modifying enzymes induced by various nanoparticles

Nanoparticles (NPs) and Nanomaterials (NMs)	In vitro or in vivo	Experimental design	Epigenetic effect	Reference
SiO_2_-NPs	In vitro	Mouse Bhas 42 cells exposed to 15 or 25 µg/cm^2^ of the crystalline silica particles Min-U-Sil^®^ 5 for 48 h	Increase in H3K4ac, H3K4me3, H3K9ac, H3K27ac Increase in HDAC2. Decrease in HDAC1, HDAC6	[22]
SiO_2_-NPs	In vitro	Human A549 cells exposed to 50.0 µg/mL SiO_2_-NPs for 3-12 h	Decreased levels of SIRT6 histone deacetylase (HDAC) transcript and protein	[63]
TiO_2_ –NPs	In vitro	Human dermal fibroblasts isolated from neonatal foreskins, exposed to 100, 30, 10, 3, and 1 µg/mL TiO_2_ -NPs for 24 h	Increase of ATM, and Chk2 phosphorylation	[54]
TiO_2_ -NPs	In vitro	Human adipose delivered stem cells (hASCs) exposed to 70 nm TiO_2_–nanotubes	Increase of H3K4 methylation at the promoter region of osteogenic genes RUNX2 and osteocalcin (OC) Inhibition of histone demethylate RBP2 expression	[64]
Au-NPs	In vitro	Human MDA-MB-231 and MDA-MsB-468 breast cancer cells exposed to 100, 250, and 500 µg/mL positively (+) and to 250 and 500 µg/mL negatively (-) charged Au-NPs for 24 h	Activation of MAP kinases in MDA-MB-231 cells Increase in MKP-1 protein in (-) AU-NPs in both cell lines Decrease in MKP-1 protein levels by (+) charged Au-NPs. Deacetylation of histone H3K9/H3K14 Dephosphorylation of histone H3Ser10 at 250 µg/mL negatively charged Au-NPs. Increase in H3K9/H3K14 acetylation at all doses of positively charged Au-NPs.	[57]
Au-NPs	In vitro	Small airway epithelial cells exposed to 20 nm Au-NPs for 72 h	Decrease in H3K27me3	[10]
Arsenic trioxide NPs (As_2_O_3_-NPs)	In vitro	Human embryonic kidney (HEK) 293T or HeLa cells were exposed to As_2_O_3_-NPs at 0.2- 0.8 µM for 24, 48, and 72 h	Decrease in global H4K16ac	[60]
Ag-NPs	In vitro	Human A549, MCF7, and HaCat cells exposed to 0.3 µg/mL Ag-NPs for 24 h	Increase in histone 3 serine 10 phosphorylation (H3S10ph)	[65]
Ag-NPs	In vitro	Human lung adenocarcinoma epithelial cells A549, exposed to 1.0 µg/mL Ag-NPs for 10 h	Increase of histone H3 serine 10 phosphorylation (H3S10ph) independent of DNA damage	[67]
Ag-NPs	In vitro	Human lung adenocarcinoma epithelial cells A549, exposed to 10-200 µg/mL Ag-NPs for 48 and 72 h	Deacetylation of histone H3 tails and elevation of total histone H3 Phosphorylation of p53	[35]
Ag-NPs	In vitro	Mouse erythroleukemia cells exposed to 8 µg/mL Ag-NPs for 72 h	Decrease in global and β-globin specific histone H3 lysine 4 trimethylation (H3Kme3) and histone H3 lysine 79 monomethylation (H3K79me1)	[68]
CuO-NPs	In vitro	Human A549 cells exposed to CuO-NPs for 36 h	Decrease of total HDAC activity. Reduction in the levels of HDAC1, HDAC2, HDAC3, HDAC5, HDAC9, and HDAC11 mRNA transcripts.	[72]
Zinc oxide nanoparticles (ZnO-NPs)	In vitro	HaCaT cells exposed to 20 and 50 µg/mL ZnO-NPs for 24 h	Deacetylation of histone H4 lysine 5 (H4K3) Increased demethylation of histone H3 lysine 9 (H3K9) Increased expression of G9a and GLP histone methyltransferase genes Down-regulation of GCN5, P300, and CBP histone acetyltransferase genes	[69]
ZnO-NPs	In vitro	Human bladder cancer T24 cells exposed to 10 µg/mL ZnO-NPs for 48 h	Decrease of global histone 3 lysine 27 trimethylation (H3K27me3) at the *RUNX3* gene promoter	[71]
Arsenic trioxide nanoparticles (As_2_O_3_-NPs)	In vitro	Human embryonic kidney (HEK) 293T or HeLa cells were exposed to As_2_O_3_-NPs at different concentrations (0.2 ~ 0.8 μM) for 24, 48, or 72 h	Reduction of global histone 4 lysine 16 acetylation (H4K16ac) Increase of deacetyltransferase HDAC4 expression	[60]
Nano-cobalt (Nano-Co) and TiO_2_ -NPs	In vitro	Human lung adenocarcinoma epithelial cells A549, exposed to 5-15 µg/mL TiO_2_ -NPs and Nano-Co for 12 h	Increased expression of Rad51, and phosphorylated p53	[52]
Cadmium telluride quantum dots (CdTe-QDs)	In vitro	Human breast cancer cells MCF-7 were exposed to 5 μg/ml CdTe-QDs for 4 or 24 h	Global histone hypoacetylation	[70]

### Silica nanoparticles

Seidel et al. [22] showed that the treatment of mouse Bhas 42 cells with 15 or 25 µg/cm^2^ of the crystalline silica particle Min-U-Sil^®^5 increased acetylation of histones H3 and H4, increased the level of HDAC2 protein, and decreased levels of HDAC1 and HDAC6 proteins. Additionally, the levels of transcription activating histone modifications, histone H4Kme3, H3K4ac, H3K9ac, and H3K27ac marks, were increased at the promoter region of the over-expressed *c*-*myc* gene. Additionally, it has been demonstrated that treatment of human A549 cells with 50 µg/mL SiO_2_-NPs for 3–12 h destabilizes histone deacetylase (HDAC) SIRT6 mRNA resulting in decreased levels of SIRT6 transcript and protein [63].

### Titanium dioxide nanoparticles

Lv et al. [64] investigated the effect of TiO_2_ nanotubes on human adipose-derived stem cells (hASCs) and reported an osteogenic differentiation of hASCs exposed to TiO_2_ nanotubes with a diameter of 70 nm. Mechanistically, the osteogenic differentiation of hASCs was associated with increased histone H3K4 methylation at the promoter region of osteogenic genes *RUNX2* and osteocalcin (*OC*) and with the inhibition of histone demethylase *RBP2* expression.

### Gold and arsenic nanoparticles

Several types of altered histone modifications have been reported upon exposure to Au-NPs. Shyamasundar et al. [10] demonstrated that exposure of small airway epithelial cells to 20 nm Au-NPs for 72 h decreased histone H3 lysine 27 trimethylation (H3K27me3), and Surapaneni et al. [57] showed that treatment of human MDA-MB-231 breast cancer cells with 250 and 500 µg/mL negatively charged Au-NPs for 24 h resulted in deacetylation of histone H3K9/H3K14 and dephosphorylation of histone H3Ser10, whereas treatment of the same cells with 100, 250 and 500 µg/mL positively charged Au-NPs caused increased H3K9/H3K14 acetylation and histone H3Ser10 phosphorylation. Liu et al. [60] reported the reduction of global histone H4K16 acetylation in human embryonic kidney HEK293 or HeLa cells exposed to arsenic trioxide NPs (As_2_O_3_-NPs) at 0.2–0.8 µM for 24, 48, and 72 h.

### Silver nanoparticles

Zhao et al. [65] demonstrated that treatment of human A549, MCF-7, and HaCaT cells with 0.3 µg/mL Ag-NPs for 24 h resulted in increased phosphorylation of histone H3 serine 10 (H3Ser10ph), a modification associated with mitotic chromatin condensation [66] and the activation of Aurora kinases. This finding was confirmed in a subsequent study showing that exposure of A549 cells to 1.0 µg/mL Ag-NPs for 10 h caused an increased level of histone H3Ser10ph that was independent of DNA damage [67]. In another study, Blanco et al. [35] reported that treatment of A549 cells with 10-200 µg/mL Ag-NPs for 48 or 72 h induced dramatic deacetylation of histone H3. A marked decrease in levels of global and β-globin gene-specific histone H3 lysine 4 trimethylation (H3K4me3) and histone H3 lysine 79 monomethylation (H3K79me1) was found in mouse erythroleukemia cells treated with 8 µg/mL Ag-NPs for 72 h [68].

### Zinc oxide nanoparticles

Gao et al. [69] demonstrated marked deacetylation of histone H4 lysine 5 (H4K5) and increased demethylation of histone H3 lysine 9 (H3K9) in HaCaT cells treated with 20 and 50 µg/mL ZnO-NPs for 24 h. Similar exposure-related global histone hypoacetylation was reported after exposure of human breast cancer cells to cadmium telluride quantum dots [70]. Recently, Zhang et al. [71] reported that treatment of human bladder cancer T24 cells with 10 µg/mL ZnO-NPs for 48 h decreased the level of global histone H3K27 trimethylation and at the *RUNX3* gene promoter. Mechanistically, these changes were attributed to the down-regulation of histone H3K27 methyltransferase EZH2. Additionally, increased expression of G9a and GLP histone methyltransferase genes and down-regulation of GCN5, P300, and CBP histone acetyltransferase genes were observed in HaCaT cells treated with ZnO-NPs [69].

### Copper oxide nanoparticles

Kalaiarasi et al. [72] reported a dramatic decrease of total HDAC activity in A549 cells treated with CuO-NPs for 36 h, which was accompanied by a reduction in the levels of *HDAC1*, *HDAC2*, *HDAC3*, *HDAC5*, and *HDAC1*1 mRNA transcripts.

## Mechanisms of DNA methylation and histone modification alterations induced by nanomaterials and nanoparticles

In normal cells, epigenetic mechanisms are well controlled and maintained (Fig. 1a) but undergo substantial abnormal alterations upon exposure to NMs and NPs resulting in aberrant expression of protein-coding and protein-non-coding genes (Fig. 1b).

**Fig. 2:**
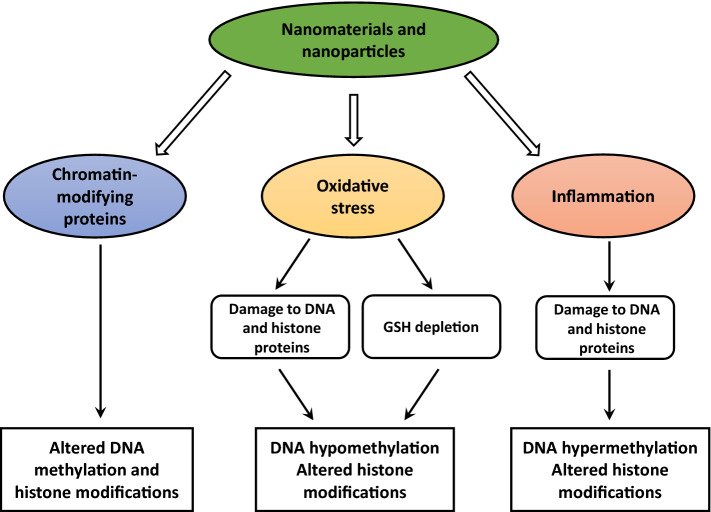
Mechanism of DNA methylation and histone modification alterations induced by nanomaterials and nanoparticles. Exposure to NMs and NPs alters the functioning of chromatin-modifying proteins, e.g., DNA methylation and demethylation machinery, and histone-modifying enzymes, causing changes in the pattern of DNA methylation and histone modifications. One of the most common effects of NMs and NPs is the induction of cellular stress, e.g., oxidative and endoplasmic reticulum stress, and metabolic disturbances, e.g., one-carbon metabolism and the citric acid cycle. These events are causing DNA damage and repair response and metabolic alterations affecting the functioning of chromatin-modifying enzymes. Any or all of these events may result in hypomethylation of DNA and altered histone modification patterns. Additionally, exposure to NMs and NPs causes activation of the inflammatory response that, in turn, may cause DNA hypermethylation and histone modification changes

The mechanism of alterations in cytosine DNA methylation and histone modifications upon exposure to nanomaterials and nanoparticles remains unclear; more than a single mechanism is very likely contributing to these changes (Fig. 2). It is well established that several factors are involved in the accurate maintenance of cytosine DNA methylation and histone modifications, including the proper function of chromatin-modifying proteins, the status of intracellular metabolism, especially one-carbon metabolism, and chromatin integrity. Many studies of DNA methylation or histone modification response to NMs and NPs exposure attempt to link observed changes in DNA methylation and histone modifications to altered functioning of chromatin-modifying proteins; however, the results of these studies are inconclusive. In contrast, one common observation specific to the majority of studies is the induction of oxidative stress and inflammation [38, 52, 53, 73]. It is well established that these two events affect the integrity of chromatin and lead to changes in DNA methylation and histone modifications [74–76]. Specifically, the presence in DNA oxidative stress-induced DNA lesions, such as 8-OHdG and 5-hmeC, inhibits methylation capacity of DNA methyltransferases, leading to global DNA hypomethylation [74], a DNA methylation change often induced by nanomaterials and nanoparticles. Additionally, the results of several independent studies have demonstrated that exposure to NMs and NPs, e.g., TiO_2_-NPs, ZnO-NPs, MWCNTs, etc., resulted in depletion of glutathione [26, 77], a main non-enzymatic cellular antioxidant, loss of which triggers a number of intracellular events leading to demethylation of DNA and histone alterations [78, 79].

Alterations in gene-specific DNA methylation also occur. This change in DNA methylation may be attributed to inflammation-induced DNA damage. The presence of inflammation-mediated halogenated cytosine damage products in DNA such as 5-chlorocytosine and 5-bromocytosine can mimic 5-meC and direct methylation to previously unmethylated CpG sites, which promotes aberrant hypermethylation [75]. Furthermore, it has been reported that oxidative DNA damage can simultaneously induce DNA demethylation and generation of new methylation sites at unmethylated CpG sites [76].

## Role of epigenetic alterations in the safety assessment of nanomaterials and nanoparticles

The field of nanotoxicology is rapidly expanding to define key events associated with toxicity of nanomaterials and nanoparticles. Accumulated evidence from experimental and epidemiological studies demonstrates that epigenetic alterations may be used to detect toxicity caused by engineered nanomaterials and nanoparticles and, more importantly, to predict their toxicity in preclinical assessments. Several in vitro and in vivo experimental studies have documented an association between toxicity and epigenetic alterations after exposure to certain nanomaterials and nanoparticles [22, 27, 64, 80]. This suggests that epigenetic alterations can be valuable indicators of nanomaterials and nanoparticles toxicity and can be potential translational biomarkers for detecting adverse effects of nanomaterials in humans. This can be illustrated by the loss of global DNA methylation found in human and mouse cells after exposure to silica nanoparticles in vitro [22, 23] and confirmed by the elegant independent study of Liou et al. [44] which showed a significant reduction in global cytosine DNA methylation in white blood cells from workers exposed to SiO_2_-NPs. In the studies presented in this review, engineered nanomaterials were extensively characterized, and their stability was tested before use in vitro or in vivo. However, despite these promising findings, a long list of unanswered questions remains. Specifically, the main limitation of many existing studies focused on the effect of nanomaterials and nanoparticles on epigenetic mechanisms, is that the causal mechanistic relationship has not been established. In particular, one of the main concurrent findings in the studies on the effects of NMs and NPs is cell toxicity. Therefore, it is not clear if NMs and NPs exposure directly affected epigenome, or the observed exposure-related changes are caused by cell toxicity. Extra caution should be taken in the interpretation of the significance and role of observed exposure-related changes in the mechanism of NMs and NPs toxicity. This is related to the fact that many experimental studies have used different cell lines or animal models, different times of exposure, various, sometimes highly toxic doses, of NMs and NPs, and different methodologies in the analysis of epigenetic alterations, ranging from simple techniques of global DNA methylation analysis to highly sophisticated array-based or next-generation sequencing technologies. Furthermore, most of the current studies in the field of epigenetic toxicology have focused on the epigenetic effects of overall NMs and NPs exposure without taking into consideration the role of different shape, charge, size, composition, and surface chemical modifications of NMs and NPs on the epigenome response. Additionally, majority of the existing studies present rather a snapshot of exposure-related epigenetic alterations. All of these make it difficult to determine the significance of epigenetic abnormalities in the mechanisms of toxicity of nanomaterials and nanoparticles, especially to distinguish the role of epigenetic alterations as one of the driver events of toxicity or just as one of the transitory non-specific cellular responses. Future well-designed and well-controlled studies are needed for better understanding of the mechanisms and processes associated with epigenetic alterations induced by the nanoparticles and nanomaterials to establish the foundation for the role of epigenetic alterations as biomarkers of nanoparticles and nanomaterials toxicity.

## Data Availability

Not applicable.

## Abbreviations

NMs: Nanomaterials
NPs: Nanoparticles
CNPs: Carbon nanoparticles
CDs: Carbon dots
M-GQDs: Modified nano-graphene quantum dots
SiO_2_-NPs: Silica dioxide nanoparticles
TiO_2_-NPs: Titanium dioxide nanoparticles
Ag-NPs: Silver nanoparticles
As_2_O_3_-NPs: Arsenic trioxide nanoparticles
ZnO-NPs: Zinc oxide nanoparticles
Au-NPs: Gold nanoparticles
CeO_2_-NPs: Cerium dioxide nanoparticles
CuO-NPs: Copper oxide nanoparticles
TET: Ten-eleven translocation
5-meC: 5-methylcytosine
5-hmeC: 5-hydroxymethylcytosine
SWCNTs: Single-walled carbon nanotubes
MWCNTs: Multi-walled carbon nanotubes
SAEC: Human small airway epithelial cells
8-OHdG: 8-hydroxy-2′-deoxyguanosine
OC: Osteocalcin
HDAC: Histone deacetylase
hASCs: Human adipose-derived stem cells

## References

[1] Gupta R, Xie H (2018). Nanoparticles in daily life: applications, toxicity and regulations. J Environ Pathol Toxicol Oncol.

[2] Nanodatabase, http://nanodb.dk/en Accessed 29 April 2020.

[3] Nanowerk, http:www.nanowerk.comnanomaterial-database.php Accessed 29 April 2020.

[4] StatNano, http://statnano.com/nanomaterials Accessed 29 April 2020.

[5] Ameh T, Sayes SM (2019). The potential exposure and hazards of copper nanoparticles: a review. Environ Toxicol Pharmacol.

[6] Missaoui WN, Arnold RD, Cummings BS (2018). Toxicological status of nanoparticles: what we know and what we don’t know. Chem Biol Interact.

[7] Schulte PA, Leso V, Niang M, Iavicoli I (2019). Current state of knowledge on the health effects of engineered nanomaterials in workers: a systematic review of human studies and epidemiological investigations. Scand J Work Environ Health.

[8] Xiaoli F, Qiyue C, Weihong G, Yaqing Z, Chen H, Junrong W, Longquan S (2020). Toxicology data of graphene-family nanomaterials: an update. Arch Toxicol.

[9] Gharpure S, Akash A, Ankamwar B (2020). A Review on antimicrobial properties of metal nanoparticles. J Nanosci Nanotechnol.

[10] Shyamasundar S, Ng CT, Yung LYL, Dheen ST, Bay BH (2015). Epigenetic mechanisms in nanomaterial-induced toxicity. Epigenomics.

[11] Sierra MI, Valdés A, Fernández AF, Torrecillas R, Fraga MF (2016). The effect of exposure to nanoparticles and nanomaterials on the mammalian epigenome. Int J Nanomedicine.

[12] Razin A, Riggs AD (1980). DNA methylation and gene function. Science.

[13] Ooi SK, O’Donnel AH, Bestor TH (2009). Mammalian cytosine methylation at a glance. J Cell Sci.

[14] Wu X, Zhang Y (2017). TET-mediated active DNA demethylation: mechanism, function and beyond. Nat Rev Genet.

[15] Öner D, Moisse M, Ghosh M, Duca R-C, Poels K, Luyts K (2017). Epigenetic effects of carbon nanotubes in human monocytic cells. Mutagenesis.

[16] Öner D, Ghosh M, Bové H, Moisse M, Boeckx B, Duca R-C (2018). Differences in MWCNT- and SWCNT-induced DNA methylation alterations in association with the nuclear deposition. Part Fibre Toxicol.

[17] Öner D, Ghosh M, Coorens R, Bové H, Moisse M, Lambrechts D (2020). Induction and recovery of CpG site specific methylation changes in human bronchial cells after long-term exposure to carbon nanotubes and asbestos. Environ Int.

[18] Ghosh M, Öner D, Duca R-C, Bekaert B, Vanoirbeek J, Godderis L (2018). Single-walled and multi-walled carbon nanotubes induce sequence-specific epigenetic alterations in 16 HBE cells. Oncotarget.

[19] Emerce E, Ghosh M, Öner D, Duca R-C, Vanoirbeek J, Bekaert B (2019). Carbon nanotube- and asbestos-induced DNA and RNA methylation changes in bronchial epithelial cells. Chem Res Toxicol.

[20] Sima M, Vrbova K, Zavodna T, Honkova K, Chvojkova I, Ambroz A, et al. The differential effect of carbon dots on gene expression and DNA methylation of human embryonic lung fibroblasts as a function of surface charge and dose. Int J Mol Sci. 2020;4763. 10.3390/ijms21134763 PMC7369946 32635498

[21] Gong C, Tao G, Yang L, Liu J, Liu Q, Zhuang Z (2010). SiO_2_ nanoparticles induce global genomic hypomethylation in HaCaT cells. Biochem Biophys Res Commun.

[22] Seidel C, Kirsch A, Fontana C, Visvikis A, Remy A, Gaté L (2017). Epigenetic changes in the early stage of silica-induced cell transformation. Nanotoxicology.

[23] Sooklert K, Nilyai S, Rojanathanes R, Jindatip D, Sae-Liang N, Kitkumthorn N (2019). N-acetylcysteine reverses the decrease of DNA methylation status caused engineered gold, silicon, and chitosan nanoparticles. Int J Nanomedicine.

[24] Gong C, Tao G, Yang L, Liu J, Liu Q, Li W (2012). Methylation of PARP-1 promoter involved in the regulation of nano-SiO_2_-induced decrease of PARP-1 mRNA expression. Toxicol Lett.

[25] Zou Y, Li Q, Jiang L, Guo C, Li Y, Yu Y (2016). DNA hypermethylation of CREB3L1 and Bcl-2 associated with the mitochondrial-mediated apoptosis via PI3K/Akt pathway in human BEAS-2B cells exposure to silica nanoparticles. PLoS ONE.

[26] Patil NA, Gade WN, Deobagkar DD (2016). Epigenetic modulation upon exposure of lung fibroblasts to TiO_2_ and ZnO nanoparticles: alterations in DNA methylation. Int J Nanomedicine.

[27] Stoccoro A, Di Bucchianico S, Coppedé F, Ponti J, Uboldi C, Blosi M (2017). Multiple endpoints to evaluate pristine and remediated titanium dioxide nanoparticles genotoxicity in lung epithelial A549 cells. Toxicol Lett.

[28] Lu X, Miousse IR, Pirela SV, Melnyk S, Koturbash I, Demokritou P (2016). Short-term exposure to engineered nanomaterials affects cellular epigenome. Nanotoxicology.

[29] Pogribna M, Koonce NA, Mathew A, Word B, Patri AK, Lyn-Cook B (2020). Effect of titanium dioxide nanoparticles on DNA methylation in multiple human cell lines. Nanotoxicology.

[30] Patil YM, Rajpathak SN, Deobagkar DD (2019). Characterization and DNA methylation modulatory activity of gold nanoparticles synthesized by *Pseudoalteromonas* strain. J Biosci.

[31] Smolkova B, Miklikova S, Horvatova KajabovaV, Babelova A, El Yamani N, Zdurencikova M, et al. Global and gene specific DNA methylation in breast cancer cells was not affected during epithelial-to-mesenchymal transition in vitro. Neoplasma. 2016;63:901-10. 10.4149/neo_2016_609 27565328

[32] Brzóska K, Grądzka I, Kruszewski M (2019). Silver, gold, and iron oxide nanoparticles alter miRNA expression but do not affect DNA methylation in HepG2 cells. Materials.

[33] Ng CT, Dheen ST, Yip W-C, Ong C-N, Bay B-H, Yung L-YL. The induction of epigenetic regulation of PROS1 gene in lung fibroblasts by gold nanoparticles and implications for potential lung injury. Biomaterials. 2011;32:7609-15. 10.1016/j.biomaterials.2011.06.038 21764123

[34] Mytych J, Zebrowski J, Lewinska A, Wnuk M (2017). Prolonged effects of silver nanoparticles on p53/p21 pathway-mediated proliferation, DNA damage response, and methylation parameters in HT22 hippocampal neuronal cells. Mol Neurobiol.

[35] Blanco J, Lafuente D, Gómez M, García T, Domingo JL, Sánchez DJ (2017). Polyvinyl pyrrolidone-coated silver nanoparticles in a human lung cancer cells: time- and dose-dependent influence over p53 and caspase-3 protein expression and epigenetic effects. Arch Toxicol.

[36] Gliga AR, Di Bucchianico S, Lindvall J, Fadeel B, Karlsson HL (2018). RNA-sequencing reveals long-term effects of silver nanoparticles on human lung cells. Sci Rep.

[37] Choudhury SR, Ordaz J, Lo CL, Damayanti NP, Zhou F, Irudayaraj J (2017). Zinc oxide nanoparticles-induced reactive oxygen species promotes multimodal cyto- and epigenetic toxicity. Toxicol Sci.

[38] Brown TA, Lee JW, Holian A, Porter V, Fredriksen H, Kim M (2016). Alterations in DNA methylation corresponding with lung inflammation and as a biomarker for disease development after MWCNT exposure. Nanotoxicology.

[39] Lu X, Miousse IR, Pirela SV, Moore JK, Melnyk S, Koturbash I (2016). vivo epigenetic effects induced by engineered nanomaterials: a case study of copper oxide and laser printer-emitted engineered nanoparticles. Nanotoxicology.

[40] Zhou W, Tian D, Je J, Yan X, Zhao J, Yuan X (2019). Prolonged exposure to carbon nanoparticles induced methylome remodeling and gene expression in zebrafish heart. J Appl Toxicol.

[41] Hu J, Lin W, Lin B, Wu K, Fan H, Yu Y (2019). Persistent DNA methylation changes in zebrafish following graphene quantum dots exposure in surface chemistry-dependent manner. Ecotoxicol Environ Saf.

[42] Tabish AM, Poels K, Byun HM, Luyts K, Baccarelli AA, Martens J (2017). Changes in DNA methylation in mouse lungs after a single intra-tracheal administration of nanomaterials. PLoS ONE.

[43] Rossner P, Vrbova K, Rossnerova A, Zavodna T, Milcova A, Klema J (2020). Gene expression and epigenetic changes in mice following inhalation of Copper(II) oxide nanoparticles. Nanomaterials.

[44] Liou S-H, Wu W-T, Liao H-Y, Chen C-Y, Tsai C-Y, Jung W-T (2017). Global DNA methylation and oxidative stress biomarkers in workers exposed to metal oxide nanoparticles. J Hazard Mater.

[45] Pilger A, Rϋdiger HW (2006). 8-Hydroxy-2′-deoxyguanosine as a marker of oxidative DNA damage related to occupational and environmental exposures. Int Arch Occup Environ Health.

[46] Ghosh M, Öner D, Poels K, Tabish AM, Vlaanderen J, Pronk A (2017). Changes in DNA methylation induced by multi-walled carbon nanotube exposure in the workplace. Nanotoxicology.

[47] Rossnerova A, Honkova K, Pelclova D, Zdimal V, Hubacek JA, Chvojkova I (2020). DNA Methylation Profiles in a Group of Workers Occupationally Exposed to Nanoparticles. Int J Mol Sci.

[48] Torres IO, Fudjimori DG (2015). Functional coupling between writers, erasers and readers of histone and DNA methylation. Curr Opin Struct Biol.

[49] Kopp B, Dario M, Zalko D, Audebert M (2018). Assessment of a panel of cellular biomarkers and the kinetics of their induction in comparing genotoxic modes of action in HepG2 cells. Environ Mol Mutagen.

[50] Zhao X, Takabayashi F, Ibuki Y (2016). Coexposure to silver nanoparticles and ultraviolet A synergistically enhances the phosphorylation of histone H2AX. J Photochem Photobiol B.

[51] Toyooka T, Amano T, Ibuki Y (2012). Titanium dioxide particles phosphorylate histone H2AX independent of ROS production. Mutat Res.

[52] Wan R, Mo Y, Feng L, Chien S, Tollerud DJ, Zhang Q (2012). DNA damage caused by metal nanoparticles: involvement of oxidative stress and activation of ATM. Chem Res Toxicol.

[53] Hanot-Roy M, Tubeuf E, Guilbert A, Bado-Nilles A, Vigneron P, Trouiller B (2016). Oxidative stress pathways involved in cytotoxicity and genotoxicity of titanium dioxide (TiO_2_) nanoparticles on cells constitutive of alveolo-capillary barrier in vitro. Toxicol In Vitro.

[54] Prasad RY, Chastain PD, Nikolaishvili-Feinberg N, Smeester L, Kaufmann WK, Fry RC (2013). Titanium dioxide nanoparticles activate the ATM-Chk2 DNA damage response in human dermal fibroblasts. Nanotoxicology.

[55] Setyawati MI, Khoo PK, Eng BH, Xiong S, Zhao X, Das GK (2013). Cytotoxic and genotoxic characterization of titanium dioxide, gadolinium oxide, and poly(lactic-co-glycolic acid) nanoparticles in human fibroblasts. J Biomed Mater Res A.

[56] Tarantini A, Lanceleur R, Mourot A, Lavault MT, Casterou G, Jarry G (2015). Toxicity, genotoxicity and proinflammatory effects of amorphous nanosilica in the human intestinal Caco-2 cell line. Toxicol In Vitro.

[57] Surapaneni SK, Bashir S, Tikoo K (2018). Gold nanoparticles-induced cytotoxicity in triple negative breast cancer involves different epigenetic alterations depending upon the surface charge. Sci Rep.

[58] Kung M-L, Hsieh S-L, Wu C-C, Chu T-H, Lin Y-C, Yeh B-W (2015). Enhanced reactive oxygen species overexpression by CuO nanoparticles in poorly differentiated hepatocellular carcinoma cells. Nanoscale.

[59] Könen-Adıgüzel S, Ergene S (2018). In vitro evaluation of the genotoxicity of CeO_2_ nanoparticles in human peripheral blood lymphocytes using cytokinesis-block micronucleus test, comet assay, and gamma H2AX. Toxicol Ind Health.

[60] Liu D, Wu D, Zhao L, Yang Y, Ding J, Dong L (2015). Arsenic trioxide reduces global histone H4 acetylation at lysine 16 through direct binding to histone acetyltransferase hMOF in human cells. PLoS ONE.

[61] Liu J, Zhao Y, Ge W, Zhang P, Liu X, Zhang W (2017). Oocyte exposure to ZnO nanoparticles inhibits early embryonic development through the γ-H2AX and NF-κB signaling pathways. Oncotarget.

[62] Fernandez-Bertólez N, Costa C, Brandăo F, Kiliç G, Duarte JA, Teixeria JP (2018). Toxicological assessment of silica-coated iron oxide nanoparticles in human astrocytes. Food Chem Toxicol.

[63] Zhang L, Han B, Xiang J, Liu K, Dong H, Gao X (2018). Silica nanoparticle releases SIRT6-induced epigenetic silencing of follistatin. Int J Biochem Cell Biol.

[64] Lv L, Liu Y, Zhang P, Zhang X, Liu J, Chen T (2015). The nanoscale geometry of TiO2 nanotubes influences the osteogenic differentiation of human adipose-derived stem cells by modulating H3K4 trimethylation. Biomaterials.

[65] Zhao X, Toyooka T, Ibuki Y (2017). Silver nanoparticle-induced phosphorylation of histone H3 at serine 10 is due to dynamic changes in actin filaments and the activation of Aurora kinases. Toxicol Lett.

[66] Johansen KM, Johansen J (2006). Regulation of chromatin structure by histone H3S10 phosphorylation. Chromosome Res.

[67] Zhao X, Rao Y, Liang J, Lin S, Wang X, Li Z (2019). Silver Nanoparticle-Induced Phosphorylation of Histone H3 at Serine 10 Involves MAPK Pathways. Biomolecules.

[68] Qian Y, Zhang J, Hu Q, Xu M, Chen Y, Hu G (2015). Silver nanoparticle-induced hemoglobin decrease involves alteration of histone 3 methylation status. Biomaterials.

[69] Gao F, Ma N, Zhou H, Wang Q, Zhang H, Wang P (2016). Zinc oxide nanoparticles-induced epigenetic change and G2/M arrest are associated with apoptosis in human epidermal keratinocytes. Int J Nanomed.

[70] Choi AO, Brown SE, Szyf M, Maysinger D (2008). Quantum dot-induced epigenetic and genotoxic changes in human breast cancer cells. J Mol Med.

[71] Zhang T, Du E, Liu Y, Cheng J, Zhang Z, Xu Y (2020). Anticancer effects of zinc oxide nanoparticles through altering the methylation status of histone on bladder cancer cells. Int J Nanomed.

[72] Kalaiarasi A, Sankar R, Anusha C, Saravanan K, Aarthy K, Karthic S (2018). Copper oxide nanoparticles induce anticancer activity in A549 lung cancer cells by inhibition of histone deacetylase. Biotechnol Lett.

[73] Dusinska M, Tulinska J, N El Yamani, Kuricova M, Liskova A, Rollerova E, et al. Immunotoxicity, genotoxicity and epigenetic toxicity of nanomaterials: New strategies for toxicity testing? Food Chem Toxicol. 2017;109 (Pt 1):797-811. 10.1016/j.fct.2017.08.030 28847762

[74] Valinluck V, Tsai HH, Rogstad DK, Burdzy A, Bird A, Sowers LC (2004). Oxidative damage to methyl-CpG sequences inhibits the binding of the methyl-CpG binding domain (MBD) of methyl-CpG binding protein 2 (MeCP2). Nucleic Acids Res.

[75] Valinluck V, Sowers LC (2007). Inflammation-mediated cytosine damage: a mechanistic link between inflammation and the epigenetic alterations in human cancers. Cancer Res.

[76] Jiang Z, Lai Y, Beaver JM, Tsegay PS, Zhao ML, Horton JK (2020). Oxidative DNA damage modulates DNA methylation pattern in human breast cancer 1 (BRCA1) gene via the crosstalk between DNA polymerase β and a *de novo* DNA methyltransferase. Cells.

[77] Long J, Ma W, Yu Z, Liu H, Cao Y (2019). Multi-walled carbon nanotubes (MWCNTs) promoted lipid accumulation in THP-1 macrophages through modulation of endoplasmic reticulum (ER) stress. Nanotoxicology.

[78] Lee D-H, Jacobs DR, Porta M (2009). Hypothesis: a unifying mechanism for nutrition and chemicals as lifelong modulators of DNA hypomethylation. Environ Health Perspect.

[79] Koturbash I, Simpson NE, Beland FA, Pogribny IP (2012). Alterations in histone H4 lysine 20 methylation: implications for cancer detection and prevention. Antioxid Redox Signal.

[80] Sargent LM, Hubbs AF, Young SH, Kashon ML, Dinu CZ, Salisbury JL (2012). Single-walled carbon nanotube-induced mitotic disruption. Mutat Res.

